# Morpholinium 2,4,6-trinitro­phenolate

**DOI:** 10.1107/S1600536808042657

**Published:** 2008-12-20

**Authors:** Nagarajan Vembu, Frank R. Fronczek

**Affiliations:** a Department of Chemistry, Urumu Dhanalakshmi College, Tiruchirappalli 620 019, India; b Department of Chemistry, Louisiana State University, Baton Rouge, LA 70803-1804, USA

## Abstract

There are two independent formula units in the asymmetric unit of the title compound, C_4_H_10_NO^+^·C_6_H_2_N_3_O_7_^−^. The morpholinium cations in both mol­ecules are puckered and adopt a chair conformation. Intermolecular N—H⋯O and C—H⋯O inter­actions generate rings of motifs *R*_2_^1^(5) and *R*_1_^2^(6). The supra­molecular aggregation is completed by the presence of two co-operative hydrogen-bonded networks of further N—H⋯O inter­actions, which generate an infinite one-dimensional chain along the base vector [100]. Two C—H⋯π interactions are also seen.

## Related literature

For a detailed account of the design of organic polar crystals, see: Pecaut & Bagieu-Beucher (1993 ▶). For hydrogen bonding in nitro­phenol complexes, see: In *et al.* (1997 ▶); Zadrenko *et al.* (1997 ▶); Mizutani *et al.* (1998 ▶). For the supra­molecular architecture of mol­ecular complexes of trinitro­phenols, see: Botoshansky *et al.* (1994 ▶); Vembu *et al.* (2003 ▶). For puckering paramaters, see: Cremer & Pople (1975 ▶). For hydrogen-bonding criteria, see: Desiraju & Steiner (1999 ▶); Desiraju (1989 ▶); Jeffrey (1997 ▶). For graph-set notation, see: Bernstein *et al.* (1995 ▶); Etter (1990 ▶).
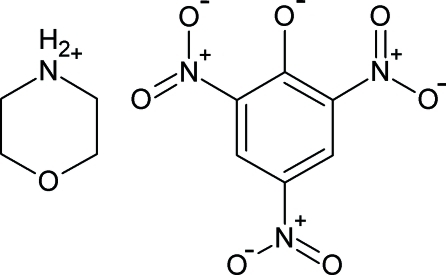


## Experimental

### 

#### Crystal data

C_4_H_10_NO^+^·C_6_H_2_N_3_O_7_^−^
*M* *_r_* = 316.24
Triclinic, 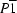

*a* = 8.3179 (5) Å
*b* = 9.5733 (5) Å
*c* = 16.8451 (10) Å
α = 91.292 (4)°
β = 98.604 (4)°
γ = 107.589 (4)°
*V* = 1261.00 (13) Å^3^
*Z* = 4
Cu *K*α radiation
μ = 1.28 mm^−1^
*T* = 90.0 (5) K
0.25 × 0.22 × 0.13 mm

#### Data collection

Bruker Kappa APEXII CCD area-detector diffractometer
Absorption correction: multi-scan (*SADABS*; Sheldrick, 2006 ▶) *T*_min_ = 0.745, *T*_max_ = 0.851
14930 measured reflections
4520 independent reflections
4172 reflections with *I* > 2σ(*I*)
*R*_int_ = 0.025

#### Refinement

*R*[*F* ^2^ > 2σ(*F* ^2^)] = 0.029
*wR*(*F* ^2^) = 0.075
*S* = 1.04
4520 reflections
494 parameters
All H-atom parameters refined
Δρ_max_ = 0.25 e Å^−3^
Δρ_min_ = −0.21 e Å^−3^

### 

Data collection: *APEX2* (Bruker, 2006 ▶); cell refinement: *APEX2* and *SAINT* (Bruker, 2006 ▶); data reduction: *SAINT*; program(s) used to solve structure: *SHELXS97* (Sheldrick, 2008 ▶); program(s) used to refine structure: *SHELXL97* (Sheldrick, 2008 ▶); molecular graphics: *PLATON* (Spek, 2003 ▶); software used to prepare material for publication: *SHELXL97*.

## Supplementary Material

Crystal structure: contains datablocks I, global. DOI: 10.1107/S1600536808042657/sj2568sup1.cif

Structure factors: contains datablocks I. DOI: 10.1107/S1600536808042657/sj2568Isup2.hkl

Additional supplementary materials:  crystallographic information; 3D view; checkCIF report

## Figures and Tables

**Table 1:** Hydrogen-bond geometry (Å, °)

*D*—H⋯*A*	*D*—H	H⋯*A*	*D*⋯*A*	*D*—H⋯*A*
N20*A*—H20*A*⋯O17*B*	0.905 (18)	1.938 (18)	2.8011 (14)	158.9 (15)
C21*A*—H21*B*⋯O13*B*	0.976 (17)	2.485 (16)	3.1491 (16)	125.1 (12)
C22*B*—H22*C*⋯O12*B*	0.985 (17)	2.531 (17)	3.3427 (16)	139.6 (12)
N20*A*—H20*B*⋯O7*B*^i^	0.930 (19)	1.885 (19)	2.6838 (14)	142.6 (15)
N20*A*—H20*B*⋯O15*B*^i^	0.930 (19)	2.225 (18)	2.9229 (15)	131.2 (14)
C18*B*—H18*C*⋯O9*B*^i^	0.954 (17)	2.559 (16)	3.3078 (17)	135.5 (12)
N20*B*—H20*C*⋯O7*A*^ii^	0.888 (18)	1.930 (18)	2.6911 (14)	142.8 (15)
N20*B*—H20*C*⋯O9*A*^ii^	0.888 (18)	2.255 (18)	2.9248 (15)	132.1 (14)
N20*B*—H20*D*⋯O12*A*^iii^	0.930 (19)	2.528 (17)	2.8693 (14)	102.0 (12)
C18*B*—H18*D*⋯O10*B*^iii^	0.967 (17)	2.571 (17)	3.1533 (16)	118.8 (12)
C21*B*—H21*C*⋯O12*A*^iii^	0.956 (16)	2.514 (16)	3.1210 (16)	121.4 (12)
N20*B*—H20*D*⋯O17*A*^iv^	0.930 (19)	1.946 (19)	2.8182 (14)	155.4 (15)
C3*B*—H3*B*⋯O16*B*^v^	0.958 (17)	2.495 (18)	3.4394 (17)	168.5 (13)
C5*A*—H5*A*⋯O10*A*^v^	0.968 (17)	2.492 (17)	3.4436 (16)	167.7 (13)
C18*A*—H18*A*⋯O15*A*^vi^	0.956 (17)	2.474 (17)	3.2628 (17)	139.7 (13)
C21*B*—H21*D*⋯O15*A*^vi^	0.965 (17)	2.461 (17)	3.3679 (16)	156.6 (12)
C21*A*—H21*A*⋯O9*B*^vii^	0.972 (16)	2.499 (16)	3.4139 (16)	156.8 (12)
C21*A*—H21*B*⋯O16*A*^viii^	0.976 (17)	2.473 (16)	3.1274 (16)	124.2 (12)
C22*A*—H22*B*⋯O13*A*^ix^	0.952 (17)	2.595 (17)	3.3916 (16)	141.3 (13)
C18*A*—H18*A*⋯*Cg*4^ix^	0.958 (18)	2.906	3.718	140.37
C22*B*—H22*C*⋯*Cg*3^x^	0.991 (18)	3.152	3.896	133.06

Symmetry codes: (i) 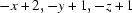; (ii) 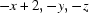; (iii) 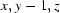; (iv) 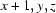; (v) 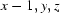; (vi) 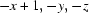; (vii) 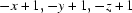; (viii) 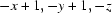; (ix) 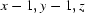; (x) 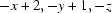. *Cg*3 and *Cg*4 are the centroids of the C1*A*–C6*A* and C1*B–*C6*B* rings, respectively.

**Fig. 1.**
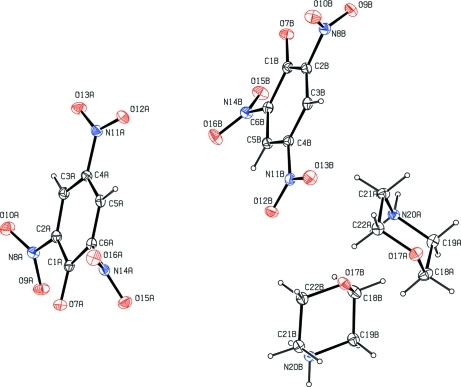
The asymmetric unit of (I) with the atoms labelled and displacement ellipsoids depicted at the 50% probability level for all non-H atoms. H-atoms are drawn as spheres of arbitrary radius.

**Fig. 2.**
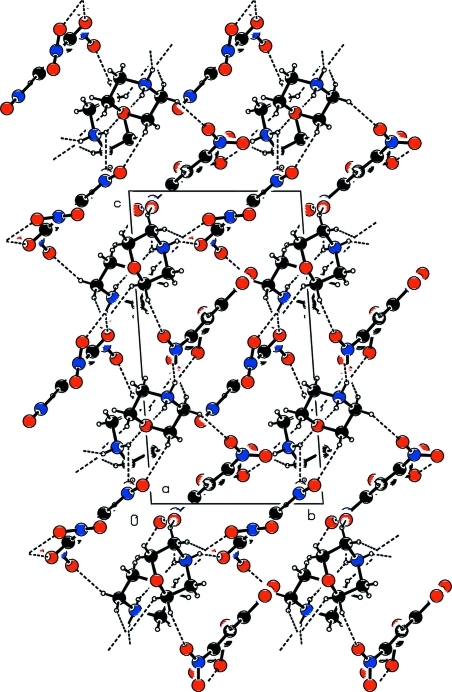
The molecular packing viewed down the a-axis. Dashed lines represent the N—H···O and C—H···O interactions within the lattice.

## supplementary crystallographic information

### Comment

The design of organic polar crystals for quadratic non-linear optical applications is supported by the observation that the organic molecules containing π-electron systems asymmetrized by electron donor and acceptor groups are highly polarizable entities in which problems of transparency and crystal growth may arise from their molecular crystal packing (Pecaut & Bagieu-Beucher, 1993). It is known that nitrophenols act not only as π-acceptor to form various π-stacking complexes with other aromatic molecules, but also as an acidic ligand to form salts through specific electrostatic or H-bonding interactions (In *et al.*, 1997). The bonding of electron-donor acceptor complexes strongly depends on the nature of the partners. The linkage could involve not only electrostatic interactions, but also the formation of molecular complexes (Zadrenko *et al.*, 1997). It has been reported that proton transferred thermochromic complexes were formed between phenols and amines in apolar solvents at low temperature if an appropriate H-bonding network between phenols and amines were present to stabilize it (Mizutani *et al.*, 1998). Pyridinium picrate has been reported in two crystalline phases and it appears in both phases as an internally linked H-bonded ion pair. These two phases are termed as molecular crystals rather than salts based on their structural arrangements (Botoshansky *et al.*, 1994). A similar structural arrangement has also been reported for 4-dimethylaminopyridinium picrate (Vembu *et al.*, 2003). In continuation of our investigations on the supramolecular architecture of picrates, the X-ray diffraction study on the title compound is carried out.

The asymmetric unit of (I) contains two morpholinium cations and and two 2,4,6-trinitrophenolate anions. (Fig.1). The morpholinium cation is puckered in both the molecules with the Cremer and Pople puckering parameters Q, θ and φ (Cremer & Pople, 1975) in the two molecules being, 0.590 (1)Å & 0.588 (1) Å, 179.7 (2)° & 0.7 (1)°, 285 (13)° & 63 (11)°, respectively. The morpolinium cation in both the molecules adopt a chair conformation as discerned from the respective torsion angles.

The crystal structure of (I) is stabilized by N—H···O and C—H···O interactions. The range of H···O distances (Table 1) found in (I) agrees with those found for N—H···O (Jeffrey, 1997) and C—H···O hydrogen bonds (Desiraju & Steiner, 1999). The N20A—H20B···O7B^i^ and N20A—H20B···O15B^i^ interactions form a pair of bifurcated donor bonds that form a motif of graph set (Bernstein *et al.*, 1995; Etter, 1990) *R*_1_^2^(6). Another pair of bifurcated donor bonds consists of the N20B—H20C···O7A^ii^ and N20B—H20C···O9A^ii^ interactions that also generate a *R*_1_^2^(6) motif. The N20B—H20D···O12A^iii^ and C21B—H21C···O12A^iii^ interactions constitute a pair of bifurcated acceptor bonds that generate a ring motif of graph set *R*_2_^1^(5). The N20A—H20A···O17B, N20A—H20B···O7B^i^ and N20A—H20B···O15B^i^ interactions generate a cooperative H-bonded network. Another cooperative H-bonded network is formed by the interactions, N20B—H20C···O7A^ii^, N20B—H20C···O9A^ii^, N20B—H20D···O12A^iii^ and N20B—H20D···O17A^iv^. These two networks generate an infinite one dimensional chain along the base vector [100]. The C18A—H18A···*Cg*4^viii^ interaction (Table 2) is classified as an offset face to face interaction with γ = 4.45° and perpendicular distance 2.897Å whereas the C22B—H22C···*Cg*3^ix^ is termed as edge to face interaction with γ = 20.34° with a perpendicular distance 2.956Å where *Cg*4 is the centroid of the ring formed by the atoms C1B—C6B and *Cg*3 is the centroid of the ring formed by the atoms C1A—C6A. There are two face to face π···π interactions in the title compound with coordinates *Cg*3···*Cg*3 (2 - *x*, 1 - *y*, -*z*) with α = 0.00°, β & γ = 19.38°, with perpendicular distance 3.449Å and *Cg*4···*Cg*4 (2 - *x*, 1 - *y*, 1 - *z*) with α = 0.02°, β & γ = 18.71°, and perpendicular distance 3.461 Å.

The interplay of strong N—H···O and weak C—H···O, C—H···π and π···π interactions with different strengths, directional preferences and distances presents a complex mosaic of interactions. The three dimensional arrangement of 2,4,6-trinitrophenolate and morpholinium moieties in the unit cell, shows that the title compound is an internally linked hydrogen bonded ion pair and hence can be regarded as a molecular crystal rather than a salt.

### Experimental

2,4,6-Trinitrophenol (5.2 mmol) dissolved in aqueous ethanol (25 ml) was added dropwise to morpholine (5.7 mmol) in aqueous ethanol (25 ml). The above solution was constantly stirred at room temperature for 2 hrs. The precipitated product was filtered and recrystallized from aqueous ethanol. Yield 75% (3.9 mmol).

### Refinement

All H-atoms were located in difference maps and their positions and isotropic displacement parameters were freely refined.

### Crystal data

**Table:**

C_4_H_10_NO^+^·C_6_H_2_N_3_O_7_^−^	*Z* = 4
*M**_r_* = 316.24	*F*(000) = 656
Triclinic, *P*1	*D*_x_ = 1.666 Mg m^−^^3^
Hall symbol: -P 1	Melting point: 418 K
*a* = 8.3179 (5) Å	Cu *K*α radiation, λ = 1.54178 Å
*b* = 9.5733 (5) Å	Cell parameters from 9038 reflections
*c* = 16.8451 (10) Å	θ = 2.6–70.3°
α = 91.292 (4)°	µ = 1.28 mm^−^^1^
β = 98.604 (4)°	*T* = 90 K
γ = 107.589 (4)°	Needle, yellow
*V* = 1261.00 (13) Å^3^	0.25 × 0.22 × 0.13 mm

### Data collection

**Table:**

Bruker Kappa APEXII CCD area-detector diffractometer	4520 independent reflections
Radiation source: fine-focus sealed tube	4172 reflections with *I* > 2σ(*I*)
graphite	*R*_int_ = 0.025
φ and ω scans	θ_max_ = 70.4°, θ_min_ = 2.7°
Absorption correction: multi-scan (*SADABS*; Sheldrick, 2006)	*h* = −9→8
*T*_min_ = 0.745, *T*_max_ = 0.851	*k* = −11→11
14930 measured reflections	*l* = −20→20

### Refinement

**Table:**

Refinement on *F*^2^	Secondary atom site location: difference Fourier map
Least-squares matrix: full	Hydrogen site location: inferred from neighbouring sites
*R*[*F*^2^ > 2σ(*F*^2^)] = 0.029	All H-atom parameters refined
*wR*(*F*^2^) = 0.075	*w* = 1/[σ^2^(*F*_o_^2^) + (0.0404*P*)^2^ + 0.5962*P*] where *P* = (*F*_o_^2^ + 2*F*_c_^2^)/3
*S* = 1.04	(Δ/σ)_max_ < 0.001
4520 reflections	Δρ_max_ = 0.25 e Å^−^^3^
494 parameters	Δρ_min_ = −0.21 e Å^−^^3^
0 restraints	Extinction correction: *SHELXL97* (Sheldrick, 2008), Fc^*^=kFc[1+0.001xFc^2^λ^3^/sin(2θ)]^-1/4^
Primary atom site location: structure-invariant direct methods	Extinction coefficient: 0.0031 (2)

### Special details

**Table:**

Geometry. All e.s.d.'s (except the e.s.d. in the dihedral angle between two l.s. planes) are estimated using the full covariance matrix. The cell e.s.d.'s are taken into account individually in the estimation of e.s.d.'s in distances, angles and torsion angles; correlations between e.s.d.'s in cell parameters are only used when they are defined by crystal symmetry. An approximate (isotropic) treatment of cell e.s.d.'s is used for estimating e.s.d.'s involving l.s. planes.
Refinement. Refinement of *F*^2^ against ALL reflections. The weighted *R*-factor *wR* and goodness of fit *S* are based on *F*^2^, conventional *R*-factors *R* are based on *F*, with *F* set to zero for negative *F*^2^. The threshold expression of *F*^2^ > σ(*F*^2^) is used only for calculating *R*-factors(gt) *etc*. and is not relevant to the choice of reflections for refinement. *R*-factors based on *F*^2^ are statistically about twice as large as those based on *F*, and *R*- factors based on ALL data will be even larger.

### Fractional atomic coordinates and isotropic or equivalent isotropic displacement parameters (Å^2^)

**Table:**

	*x*	*y*	*z*	*U*_iso_*/*U*_eq_	
C1A	1.09611 (17)	0.49974 (13)	−0.12320 (7)	0.0127 (3)	
C2A	1.25008 (16)	0.60126 (14)	−0.07769 (7)	0.0132 (3)	
C3A	1.25234 (17)	0.72107 (14)	−0.03028 (7)	0.0136 (3)	
C4A	1.10041 (17)	0.74600 (14)	−0.02152 (7)	0.0133 (3)	
C5A	0.94390 (17)	0.65574 (14)	−0.06295 (7)	0.0132 (3)	
C6A	0.94588 (16)	0.53913 (14)	−0.11109 (7)	0.0130 (3)	
O7A	1.09018 (12)	0.39624 (10)	−0.17139 (5)	0.0155 (2)	
N8A	1.41454 (14)	0.58076 (12)	−0.08198 (6)	0.0147 (2)	
O9A	1.42204 (12)	0.45459 (10)	−0.09063 (6)	0.0192 (2)	
O10A	1.54125 (12)	0.69100 (10)	−0.07467 (6)	0.0212 (2)	
N11A	1.10530 (14)	0.86621 (12)	0.03319 (6)	0.0143 (2)	
O12A	0.96928 (12)	0.87274 (10)	0.05109 (5)	0.0179 (2)	
O13A	1.24520 (12)	0.95709 (10)	0.05934 (6)	0.0194 (2)	
N14A	0.78244 (14)	0.44885 (12)	−0.15723 (6)	0.0134 (2)	
O15A	0.74323 (12)	0.31494 (10)	−0.15316 (6)	0.0189 (2)	
O16A	0.69331 (12)	0.51112 (11)	−0.19769 (6)	0.0199 (2)	
O17A	0.10539 (11)	0.00176 (10)	0.25016 (6)	0.0168 (2)	
C18A	0.20658 (18)	−0.08501 (15)	0.28608 (9)	0.0184 (3)	
C19A	0.32219 (18)	−0.00503 (15)	0.36195 (8)	0.0171 (3)	
N20A	0.43482 (14)	0.13842 (12)	0.34067 (7)	0.0133 (2)	
C21A	0.32929 (17)	0.22701 (14)	0.30180 (8)	0.0145 (3)	
C22A	0.21200 (17)	0.13892 (15)	0.22823 (8)	0.0162 (3)	
C1B	1.20643 (17)	0.72087 (13)	0.48672 (7)	0.0131 (3)	
C2B	1.02460 (17)	0.69732 (14)	0.46441 (7)	0.0136 (3)	
C3B	0.91624 (17)	0.59563 (14)	0.40640 (7)	0.0133 (3)	
C4B	0.98544 (17)	0.50853 (14)	0.36284 (7)	0.0133 (3)	
C5B	1.15875 (17)	0.52696 (14)	0.37604 (7)	0.0134 (3)	
C6B	1.26371 (16)	0.62652 (14)	0.43659 (8)	0.0134 (3)	
O7B	1.30043 (12)	0.81736 (10)	0.53895 (5)	0.0166 (2)	
N8B	0.95032 (14)	0.79251 (12)	0.50610 (6)	0.0141 (2)	
O9B	0.97719 (12)	0.80272 (10)	0.57994 (5)	0.0181 (2)	
O10B	0.86456 (13)	0.85646 (11)	0.46437 (6)	0.0208 (2)	
N11B	0.87435 (14)	0.39640 (12)	0.30331 (6)	0.0143 (2)	
O12B	0.93987 (12)	0.32440 (10)	0.26417 (5)	0.0172 (2)	
O13B	0.71918 (12)	0.37736 (11)	0.29419 (6)	0.0204 (2)	
N14B	1.44400 (14)	0.63651 (12)	0.44848 (6)	0.0152 (2)	
O15B	1.52754 (12)	0.66652 (11)	0.51713 (6)	0.0196 (2)	
O16B	1.50511 (12)	0.61178 (11)	0.38943 (6)	0.0214 (2)	
O17B	0.59893 (12)	0.00091 (10)	0.24493 (5)	0.0160 (2)	
C18B	0.71842 (18)	−0.05139 (15)	0.29696 (8)	0.0170 (3)	
C19B	0.72407 (18)	−0.19629 (15)	0.26204 (8)	0.0160 (3)	
N20B	0.77466 (14)	−0.17515 (12)	0.18046 (6)	0.0132 (2)	
C21B	0.65320 (17)	−0.11684 (14)	0.12743 (8)	0.0144 (3)	
C22B	0.64834 (18)	0.02484 (15)	0.16719 (8)	0.0157 (3)	
H3A	1.360 (2)	0.7844 (18)	−0.0031 (10)	0.017 (4)*	
H5A	0.838 (2)	0.6748 (17)	−0.0584 (9)	0.016 (4)*	
H18A	0.271 (2)	−0.1092 (17)	0.2483 (10)	0.018 (4)*	
H18B	0.126 (2)	−0.1764 (19)	0.2992 (10)	0.023 (4)*	
H19A	0.396 (2)	−0.0592 (18)	0.3852 (10)	0.018 (4)*	
H19B	0.261 (2)	0.0171 (17)	0.4011 (10)	0.017 (4)*	
H20A	0.500 (2)	0.1174 (18)	0.3066 (10)	0.019 (4)*	
H20B	0.508 (2)	0.1873 (19)	0.3868 (11)	0.025 (4)*	
H21A	0.263 (2)	0.2475 (16)	0.3409 (9)	0.012 (4)*	
H21B	0.405 (2)	0.3182 (18)	0.2864 (9)	0.018 (4)*	
H22A	0.135 (2)	0.1928 (17)	0.2053 (9)	0.015 (4)*	
H22B	0.276 (2)	0.1212 (17)	0.1890 (10)	0.017 (4)*	
H3B	0.797 (2)	0.5873 (17)	0.3967 (10)	0.017 (4)*	
H5B	1.207 (2)	0.4711 (17)	0.3456 (10)	0.014 (4)*	
H18C	0.827 (2)	0.0221 (17)	0.3037 (9)	0.014 (4)*	
H18D	0.680 (2)	−0.0648 (18)	0.3484 (10)	0.020 (4)*	
H19C	0.809 (2)	−0.2287 (17)	0.2940 (10)	0.017 (4)*	
H19D	0.616 (2)	−0.2714 (18)	0.2553 (10)	0.019 (4)*	
H20C	0.780 (2)	−0.260 (2)	0.1599 (10)	0.022 (4)*	
H20D	0.885 (2)	−0.109 (2)	0.1881 (10)	0.024 (4)*	
H21C	0.689 (2)	−0.1002 (17)	0.0762 (10)	0.015 (4)*	
H21D	0.543 (2)	−0.1912 (17)	0.1218 (9)	0.014 (4)*	
H22C	0.760 (2)	0.1022 (18)	0.1722 (9)	0.018 (4)*	
H22D	0.560 (2)	0.0609 (17)	0.1341 (10)	0.016 (4)*	

### Atomic displacement parameters (Å^2^)

**Table:**

	*U*^11^	*U*^22^	*U*^33^	*U*^12^	*U*^13^	*U*^23^
C1A	0.0151 (7)	0.0122 (6)	0.0111 (6)	0.0043 (5)	0.0024 (5)	0.0033 (5)
C2A	0.0119 (7)	0.0151 (6)	0.0135 (6)	0.0050 (5)	0.0031 (5)	0.0029 (5)
C3A	0.0138 (7)	0.0142 (6)	0.0116 (6)	0.0027 (5)	0.0018 (5)	0.0023 (5)
C4A	0.0165 (7)	0.0122 (6)	0.0114 (6)	0.0045 (5)	0.0029 (5)	0.0006 (5)
C5A	0.0133 (7)	0.0150 (6)	0.0123 (6)	0.0050 (5)	0.0040 (5)	0.0036 (5)
C6A	0.0118 (7)	0.0143 (6)	0.0117 (6)	0.0022 (5)	0.0017 (5)	0.0024 (5)
O7A	0.0157 (5)	0.0145 (4)	0.0162 (4)	0.0054 (4)	0.0012 (4)	−0.0020 (3)
N8A	0.0128 (6)	0.0155 (5)	0.0153 (5)	0.0042 (4)	0.0013 (4)	−0.0010 (4)
O9A	0.0166 (5)	0.0165 (5)	0.0254 (5)	0.0081 (4)	0.0005 (4)	−0.0028 (4)
O10A	0.0123 (5)	0.0175 (5)	0.0313 (5)	0.0006 (4)	0.0049 (4)	−0.0021 (4)
N11A	0.0162 (6)	0.0144 (5)	0.0129 (5)	0.0053 (4)	0.0027 (4)	0.0010 (4)
O12A	0.0177 (5)	0.0205 (5)	0.0184 (5)	0.0081 (4)	0.0075 (4)	0.0000 (4)
O13A	0.0166 (5)	0.0169 (5)	0.0210 (5)	0.0016 (4)	0.0003 (4)	−0.0051 (4)
N14A	0.0122 (6)	0.0152 (5)	0.0127 (5)	0.0035 (4)	0.0031 (4)	−0.0003 (4)
O15A	0.0174 (5)	0.0138 (5)	0.0227 (5)	0.0013 (4)	0.0027 (4)	−0.0010 (4)
O16A	0.0151 (5)	0.0239 (5)	0.0201 (5)	0.0073 (4)	−0.0019 (4)	0.0037 (4)
O17A	0.0115 (5)	0.0159 (5)	0.0202 (5)	0.0018 (4)	−0.0005 (4)	0.0002 (4)
C18A	0.0145 (7)	0.0140 (6)	0.0254 (7)	0.0033 (5)	0.0014 (6)	−0.0002 (5)
C19A	0.0150 (7)	0.0168 (6)	0.0193 (7)	0.0043 (5)	0.0027 (6)	0.0043 (5)
N20A	0.0124 (6)	0.0141 (5)	0.0130 (5)	0.0041 (4)	0.0014 (5)	−0.0013 (4)
C21A	0.0126 (7)	0.0139 (6)	0.0173 (6)	0.0051 (5)	0.0020 (5)	−0.0001 (5)
C22A	0.0138 (7)	0.0186 (7)	0.0156 (6)	0.0044 (5)	0.0015 (5)	0.0019 (5)
C1B	0.0146 (7)	0.0130 (6)	0.0117 (6)	0.0041 (5)	0.0021 (5)	0.0031 (5)
C2B	0.0150 (7)	0.0142 (6)	0.0127 (6)	0.0056 (5)	0.0039 (5)	0.0024 (5)
C3B	0.0132 (7)	0.0151 (6)	0.0120 (6)	0.0046 (5)	0.0027 (5)	0.0042 (5)
C4B	0.0129 (7)	0.0130 (6)	0.0120 (6)	0.0016 (5)	0.0013 (5)	0.0009 (5)
C5B	0.0162 (7)	0.0133 (6)	0.0122 (6)	0.0054 (5)	0.0044 (5)	0.0021 (5)
C6B	0.0117 (7)	0.0145 (6)	0.0142 (6)	0.0037 (5)	0.0030 (5)	0.0032 (5)
O7B	0.0156 (5)	0.0167 (5)	0.0160 (4)	0.0048 (4)	−0.0013 (4)	−0.0037 (4)
N8B	0.0123 (6)	0.0140 (5)	0.0154 (5)	0.0034 (4)	0.0022 (4)	0.0004 (4)
O9B	0.0205 (5)	0.0218 (5)	0.0126 (4)	0.0068 (4)	0.0041 (4)	−0.0011 (4)
O10B	0.0216 (5)	0.0218 (5)	0.0216 (5)	0.0128 (4)	−0.0010 (4)	0.0004 (4)
N11B	0.0143 (6)	0.0143 (5)	0.0131 (5)	0.0021 (4)	0.0031 (4)	0.0020 (4)
O12B	0.0206 (5)	0.0164 (5)	0.0148 (4)	0.0062 (4)	0.0032 (4)	−0.0021 (3)
O13B	0.0117 (5)	0.0231 (5)	0.0226 (5)	0.0006 (4)	0.0018 (4)	−0.0030 (4)
N14B	0.0140 (6)	0.0161 (5)	0.0154 (5)	0.0046 (4)	0.0020 (4)	−0.0006 (4)
O15B	0.0161 (5)	0.0258 (5)	0.0164 (5)	0.0091 (4)	−0.0037 (4)	−0.0045 (4)
O16B	0.0155 (5)	0.0316 (6)	0.0183 (5)	0.0077 (4)	0.0060 (4)	−0.0025 (4)
O17B	0.0165 (5)	0.0209 (5)	0.0137 (4)	0.0101 (4)	0.0030 (4)	0.0005 (3)
C18B	0.0163 (7)	0.0227 (7)	0.0136 (6)	0.0092 (6)	0.0008 (5)	−0.0006 (5)
C19B	0.0155 (7)	0.0190 (7)	0.0152 (6)	0.0074 (6)	0.0035 (5)	0.0030 (5)
N20B	0.0120 (6)	0.0127 (5)	0.0147 (5)	0.0037 (5)	0.0025 (4)	−0.0015 (4)
C21B	0.0115 (7)	0.0180 (6)	0.0129 (6)	0.0040 (5)	0.0010 (5)	0.0004 (5)
C22B	0.0160 (7)	0.0176 (6)	0.0149 (6)	0.0068 (6)	0.0034 (5)	0.0025 (5)

### Geometric parameters (Å, °)

**Table:**

C1A—O7A	1.2511 (16)	C1B—O7B	1.2489 (16)
C1A—C2A	1.4466 (19)	C1B—C2B	1.4490 (19)
C1A—C6A	1.4502 (18)	C1B—C6B	1.4498 (18)
C2A—C3A	1.3765 (18)	C2B—C3B	1.3705 (19)
C2A—N8A	1.4513 (17)	C2B—N8B	1.4652 (16)
C3A—C4A	1.3829 (19)	C3B—C4B	1.3978 (18)
C3A—H3A	0.957 (17)	C3B—H3B	0.958 (17)
C4A—C5A	1.3963 (19)	C4B—C5B	1.3811 (19)
C4A—N11A	1.4449 (16)	C4B—N11B	1.4445 (17)
C5A—C6A	1.3705 (18)	C5B—C6B	1.3777 (18)
C5A—H5A	0.968 (17)	C5B—H5B	0.943 (16)
C6A—N14A	1.4638 (17)	C6B—N14B	1.4563 (17)
N8A—O10A	1.2340 (15)	N8B—O9B	1.2266 (14)
N8A—O9A	1.2347 (14)	N8B—O10B	1.2292 (15)
N11A—O12A	1.2324 (15)	N11B—O13B	1.2328 (15)
N11A—O13A	1.2339 (15)	N11B—O12B	1.2333 (14)
N14A—O16A	1.2262 (15)	N14B—O16B	1.2318 (15)
N14A—O15A	1.2298 (14)	N14B—O15B	1.2356 (15)
O17A—C18A	1.4343 (16)	O17B—C22B	1.4332 (15)
O17A—C22A	1.4363 (16)	O17B—C18B	1.4376 (16)
C18A—C19A	1.5126 (19)	C18B—C19B	1.5110 (18)
C18A—H18A	0.956 (17)	C18B—H18C	0.954 (17)
C18A—H18B	0.982 (18)	C18B—H18D	0.967 (17)
C19A—N20A	1.4957 (17)	C19B—N20B	1.4968 (16)
C19A—H19A	0.964 (17)	C19B—H19C	0.952 (17)
C19A—H19B	0.949 (17)	C19B—H19D	0.957 (17)
N20A—C21A	1.4905 (16)	N20B—C21B	1.4912 (17)
N20A—H20A	0.905 (18)	N20B—H20C	0.888 (18)
N20A—H20B	0.930 (19)	N20B—H20D	0.930 (19)
C21A—C22A	1.5141 (18)	C21B—C22B	1.5134 (18)
C21A—H21A	0.972 (16)	C21B—H21C	0.956 (16)
C21A—H21B	0.976 (17)	C21B—H21D	0.965 (17)
C22A—H22A	0.977 (16)	C22B—H22C	0.985 (17)
C22A—H22B	0.952 (17)	C22B—H22D	1.003 (17)
			
O7A—C1A—C2A	125.64 (12)	O7B—C1B—C2B	123.19 (12)
O7A—C1A—C6A	122.87 (12)	O7B—C1B—C6B	125.55 (12)
C2A—C1A—C6A	111.33 (11)	C2B—C1B—C6B	111.10 (11)
C3A—C2A—C1A	123.85 (12)	C3B—C2B—C1B	125.69 (12)
C3A—C2A—N8A	116.39 (11)	C3B—C2B—N8B	116.95 (12)
C1A—C2A—N8A	119.75 (11)	C1B—C2B—N8B	117.34 (11)
C2A—C3A—C4A	119.83 (12)	C2B—C3B—C4B	118.12 (12)
C2A—C3A—H3A	118.7 (10)	C2B—C3B—H3B	119.4 (9)
C4A—C3A—H3A	121.5 (10)	C4B—C3B—H3B	122.5 (10)
C3A—C4A—C5A	121.30 (12)	C5B—C4B—C3B	121.14 (12)
C3A—C4A—N11A	118.73 (12)	C5B—C4B—N11B	119.13 (11)
C5A—C4A—N11A	119.96 (11)	C3B—C4B—N11B	119.72 (12)
C6A—C5A—C4A	117.54 (12)	C6B—C5B—C4B	119.39 (12)
C6A—C5A—H5A	120.9 (9)	C6B—C5B—H5B	118.8 (10)
C4A—C5A—H5A	121.6 (9)	C4B—C5B—H5B	121.8 (10)
C5A—C6A—C1A	126.08 (12)	C5B—C6B—C1B	124.41 (12)
C5A—C6A—N14A	117.30 (11)	C5B—C6B—N14B	116.21 (11)
C1A—C6A—N14A	116.57 (11)	C1B—C6B—N14B	119.37 (11)
O10A—N8A—O9A	123.17 (11)	O9B—N8B—O10B	124.48 (11)
O10A—N8A—C2A	117.99 (10)	O9B—N8B—C2B	118.06 (10)
O9A—N8A—C2A	118.82 (10)	O10B—N8B—C2B	117.46 (10)
O12A—N11A—O13A	123.45 (11)	O13B—N11B—O12B	123.32 (11)
O12A—N11A—C4A	118.20 (11)	O13B—N11B—C4B	118.51 (11)
O13A—N11A—C4A	118.35 (11)	O12B—N11B—C4B	118.17 (11)
O16A—N14A—O15A	124.03 (11)	O16B—N14B—O15B	123.11 (11)
O16A—N14A—C6A	118.12 (10)	O16B—N14B—C6B	118.08 (11)
O15A—N14A—C6A	117.85 (10)	O15B—N14B—C6B	118.80 (10)
C18A—O17A—C22A	110.98 (10)	C22B—O17B—C18B	111.02 (10)
O17A—C18A—C19A	110.48 (11)	O17B—C18B—C19B	110.24 (11)
O17A—C18A—H18A	110.1 (10)	O17B—C18B—H18C	108.1 (9)
C19A—C18A—H18A	111.4 (10)	C19B—C18B—H18C	111.9 (9)
O17A—C18A—H18B	106.4 (10)	O17B—C18B—H18D	107.6 (10)
C19A—C18A—H18B	109.9 (10)	C19B—C18B—H18D	109.5 (10)
H18A—C18A—H18B	108.4 (14)	H18C—C18B—H18D	109.4 (13)
N20A—C19A—C18A	108.48 (11)	N20B—C19B—C18B	108.76 (11)
N20A—C19A—H19A	107.1 (10)	N20B—C19B—H19C	106.8 (9)
C18A—C19A—H19A	111.6 (10)	C18B—C19B—H19C	111.0 (9)
N20A—C19A—H19B	106.8 (10)	N20B—C19B—H19D	107.1 (10)
C18A—C19A—H19B	112.8 (10)	C18B—C19B—H19D	112.9 (10)
H19A—C19A—H19B	109.8 (14)	H19C—C19B—H19D	110.0 (14)
C21A—N20A—C19A	110.29 (10)	C21B—N20B—C19B	110.35 (10)
C21A—N20A—H20A	110.8 (10)	C21B—N20B—H20C	112.0 (11)
C19A—N20A—H20A	107.0 (10)	C19B—N20B—H20C	109.3 (11)
C21A—N20A—H20B	112.2 (11)	C21B—N20B—H20D	111.0 (11)
C19A—N20A—H20B	109.0 (11)	C19B—N20B—H20D	106.7 (11)
H20A—N20A—H20B	107.4 (15)	H20C—N20B—H20D	107.3 (15)
N20A—C21A—C22A	108.92 (10)	N20B—C21B—C22B	109.01 (10)
N20A—C21A—H21A	107.5 (9)	N20B—C21B—H21C	109.5 (10)
C22A—C21A—H21A	110.5 (9)	C22B—C21B—H21C	110.5 (9)
N20A—C21A—H21B	109.1 (10)	N20B—C21B—H21D	106.1 (9)
C22A—C21A—H21B	110.3 (9)	C22B—C21B—H21D	110.9 (9)
H21A—C21A—H21B	110.6 (13)	H21C—C21B—H21D	110.7 (13)
O17A—C22A—C21A	110.50 (11)	O17B—C22B—C21B	110.54 (10)
O17A—C22A—H22A	106.6 (9)	O17B—C22B—H22C	109.9 (9)
C21A—C22A—H22A	109.2 (9)	C21B—C22B—H22C	111.5 (9)
O17A—C22A—H22B	109.8 (10)	O17B—C22B—H22D	107.0 (9)
C21A—C22A—H22B	110.8 (10)	C21B—C22B—H22D	109.7 (9)
H22A—C22A—H22B	109.8 (13)	H22C—C22B—H22D	108.1 (13)
			
O7A—C1A—C2A—C3A	174.43 (12)	O7B—C1B—C2B—C3B	178.42 (12)
C6A—C1A—C2A—C3A	−1.07 (17)	C6B—C1B—C2B—C3B	2.76 (18)
O7A—C1A—C2A—N8A	−4.32 (19)	O7B—C1B—C2B—N8B	−0.12 (18)
C6A—C1A—C2A—N8A	−179.83 (10)	C6B—C1B—C2B—N8B	−175.78 (10)
C1A—C2A—C3A—C4A	2.82 (19)	C1B—C2B—C3B—C4B	−1.76 (19)
N8A—C2A—C3A—C4A	−178.39 (11)	N8B—C2B—C3B—C4B	176.79 (11)
C2A—C3A—C4A—C5A	−3.08 (19)	C2B—C3B—C4B—C5B	−1.87 (18)
C2A—C3A—C4A—N11A	175.50 (11)	C2B—C3B—C4B—N11B	177.69 (11)
C3A—C4A—C5A—C6A	1.64 (18)	C3B—C4B—C5B—C6B	4.11 (19)
N11A—C4A—C5A—C6A	−176.93 (11)	N11B—C4B—C5B—C6B	−175.46 (11)
C4A—C5A—C6A—C1A	0.14 (19)	C4B—C5B—C6B—C1B	−2.93 (19)
C4A—C5A—C6A—N14A	−177.27 (11)	C4B—C5B—C6B—N14B	178.15 (11)
O7A—C1A—C6A—C5A	−176.08 (12)	O7B—C1B—C6B—C5B	−175.89 (12)
C2A—C1A—C6A—C5A	−0.42 (18)	C2B—C1B—C6B—C5B	−0.36 (17)
O7A—C1A—C6A—N14A	1.35 (18)	O7B—C1B—C6B—N14B	3.00 (19)
C2A—C1A—C6A—N14A	177.01 (10)	C2B—C1B—C6B—N14B	178.53 (10)
C3A—C2A—N8A—O10A	−30.04 (16)	C3B—C2B—N8B—O9B	128.96 (12)
C1A—C2A—N8A—O10A	148.81 (12)	C1B—C2B—N8B—O9B	−52.38 (15)
C3A—C2A—N8A—O9A	148.13 (12)	C3B—C2B—N8B—O10B	−50.82 (16)
C1A—C2A—N8A—O9A	−33.03 (17)	C1B—C2B—N8B—O10B	127.85 (12)
C3A—C4A—N11A—O12A	−167.89 (11)	C5B—C4B—N11B—O13B	177.35 (11)
C5A—C4A—N11A—O12A	10.72 (17)	C3B—C4B—N11B—O13B	−2.22 (17)
C3A—C4A—N11A—O13A	12.32 (17)	C5B—C4B—N11B—O12B	−2.60 (17)
C5A—C4A—N11A—O13A	−169.08 (11)	C3B—C4B—N11B—O12B	177.83 (11)
C5A—C6A—N14A—O16A	50.61 (16)	C5B—C6B—N14B—O16B	30.79 (16)
C1A—C6A—N14A—O16A	−127.06 (12)	C1B—C6B—N14B—O16B	−148.19 (12)
C5A—C6A—N14A—O15A	−129.58 (12)	C5B—C6B—N14B—O15B	−147.88 (12)
C1A—C6A—N14A—O15A	52.76 (15)	C1B—C6B—N14B—O15B	33.14 (17)
C22A—O17A—C18A—C19A	60.91 (14)	C22B—O17B—C18B—C19B	−61.09 (14)
O17A—C18A—C19A—N20A	−58.84 (14)	O17B—C18B—C19B—N20B	58.66 (14)
C18A—C19A—N20A—C21A	57.62 (14)	C18B—C19B—N20B—C21B	−57.23 (14)
C19A—N20A—C21A—C22A	−57.29 (13)	C19B—N20B—C21B—C22B	56.75 (13)
C18A—O17A—C22A—C21A	−60.32 (14)	C18B—O17B—C22B—C21B	60.61 (14)
N20A—C21A—C22A—O17A	57.97 (14)	N20B—C21B—C22B—O17B	−57.89 (14)

### Hydrogen-bond geometry (Å, °)

**Table:**

*D*—H···*A*	*D*—H	H···*A*	*D*···*A*	*D*—H···*A*
N20A—H20A···O17B	0.905 (18)	1.938 (18)	2.8011 (14)	158.9 (15)
C21A—H21B···O13B	0.976 (17)	2.485 (16)	3.1491 (16)	125.1 (12)
C22B—H22C···O12B	0.985 (17)	2.531 (17)	3.3427 (16)	139.6 (12)
N20A—H20B···O7B^i^	0.930 (19)	1.885 (19)	2.6838 (14)	142.6 (15)
N20A—H20B···O15B^i^	0.930 (19)	2.225 (18)	2.9229 (15)	131.2 (14)
C18B—H18C···O9B^i^	0.954 (17)	2.559 (16)	3.3078 (17)	135.5 (12)
N20B—H20C···O7A^ii^	0.888 (18)	1.930 (18)	2.6911 (14)	142.8 (15)
N20B—H20C···O9A^ii^	0.888 (18)	2.255 (18)	2.9248 (15)	132.1 (14)
N20B—H20D···O12A^iii^	0.930 (19)	2.528 (17)	2.8693 (14)	102.0 (12)
C18B—H18D···O10B^iii^	0.967 (17)	2.571 (17)	3.1533 (16)	118.8 (12)
C21B—H21C···O12A^iii^	0.956 (16)	2.514 (16)	3.1210 (16)	121.4 (12)
N20B—H20D···O17A^iv^	0.930 (19)	1.946 (19)	2.8182 (14)	155.4 (15)
C3B—H3B···O16B^v^	0.958 (17)	2.495 (18)	3.4394 (17)	168.5 (13)
C5A—H5A···O10A^v^	0.968 (17)	2.492 (17)	3.4436 (16)	167.7 (13)
C18A—H18A···O15A^vi^	0.956 (17)	2.474 (17)	3.2628 (17)	139.7 (13)
C21B—H21D···O15A^vi^	0.965 (17)	2.461 (17)	3.3679 (16)	156.6 (12)
C21A—H21A···O9B^vii^	0.972 (16)	2.499 (16)	3.4139 (16)	156.8 (12)
C21A—H21B···O16A^viii^	0.976 (17)	2.473 (16)	3.1274 (16)	124.2 (12)
C22A—H22B···O13A^ix^	0.952 (17)	2.595 (17)	3.3916 (16)	141.3 (13)
C18A—H18A···Cg4^ix^	0.958 (18)	2.906	3.718	140.37
C22B—H22C···Cg3^x^	0.991 (18)	3.152	3.896	133.06

Symmetry codes: (i) −*x*+2, −*y*+1, −*z*+1; (ii) −*x*+2, −*y*, −*z*; (iii) *x*, *y*−1, *z*; (iv) *x*+1, *y*, *z*; (v) *x*−1, *y*, *z*; (vi) −*x*+1, −*y*, −*z*; (vii) −*x*+1, −*y*+1, −*z*+1; (viii) −*x*+1, −*y*+1, −*z*; (ix) *x*−1, *y*−1, *z*; (x) −*x*+2, −*y*+1, −*z*.
